# Geometrically Constrained Growth Factor Concentration Favors Enrichment of Goblet Cells and Mucus Formation

**DOI:** 10.1021/acsbiomaterials.5c02107

**Published:** 2026-04-09

**Authors:** Cecilia Villegas-Novoa, Yuli Wang, Hao Wang, Ian Jan, Christopher E. Sims, Nancy L. Allbritton

**Affiliations:** † Department of Bioengineering, 7284 University of Washington, Seattle, Washington 98195, United States; ‡ Department of Medicine, University of Washington, Seattle, Washington 98195, United States

**Keywords:** microphysiological system, intestine-on-chip, mucus, goblet cell, large intestine, bacterial coculture, crypts

## Abstract

Colonic mucus forms a critical barrier to intestinal contents, providing the protection necessary for intestinal and organismal health. The mucus is composed of gel-forming mucin secreted by goblet cells residing in the epithelial layer lining the colon; yet, our knowledge of many of the attributes and functions of mucus and the goblet cells remains limited. A planar array of colonic cryptlike structures with a thick covering of goblet cell-generated mucus was developed to mimic the differentiated colonic epithelium and provide an easily accessible physiologic mucus layer for the evaluation of mucus barrier function in response to intestinal microbiota and toxins. The human microphysiological system (MPS) was created using an impermeable thin film patterned with a geometrical array of a 10 μm × 10 μm scale through holes overlaid with collagen and primary colonic stem cells. The array dimensions, collagen thickness, and growth factor concentration were optimized to assess the cell density, proliferation, migration, differentiation, and mucus thickness. A 175 μm center-to-center distance between the through holes or stem cell niches and a collagen thickness of 10 μm were found to be optimal to enable long-term culture (≥23 days) with a discrete stem/proliferative cell region and a differentiated cell zone enriched in goblet cells and supporting a 250 μm-thick adherent mucus layer. The mucus layer acted as an effective barrier to block the access of the *Staphylococcus aureus* α-hemolysin toxin to the epithelial cells as well as to protect the cell layer from both *Staphylococcus aureus* and *Lactobacillus rhamnosus*. The intestinal mucus MPS will be a useful tool for emulating the intestinal epithelium to study the interplay of stem cell renewal, goblet cell differentiation, mucus dynamics, and microbiota–mucus–host interactions.

## Graphical abstract

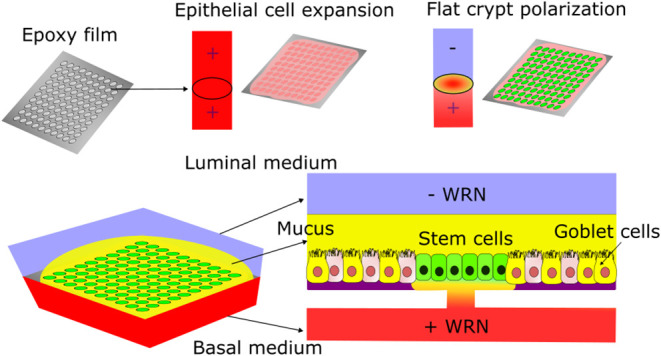

## 1 Introduction

Intestinal goblet cells continually replenish the mucus layer that serves as a barrier in the intestine to protect the epithelium of the host against native microbiota as well as pathogens and their products, e.g., toxins.^1^ The dysregulated secretion and composition of the mucus can provoke goblet cell exhaustion and defects in the quality and thickness of the mucus, allowing microbial infiltration and intestinal inflammation.^2^ Alteration in the permeability of the mucus network and goblet cell dysfunction are known to occur in intestinal diseases, most notably, in inflammatory bowel disease (IBD), Crohn’s disease (CD), and ulcerative colitis (UC).^3^ Thus, the ability to study gastrointestinal mucus and its production in a human system is essential for understanding the role of mucus in health and disease.^4,5^ The mucus layer acts as a critical barrier that can influence the absorption and bioavailability of orally administered drugs.^6^ Understanding the interaction between drugs and mucus has led to the development of novel formulations that enhance drug penetration and stability in the gastrointestinal tract.^7,8^ Furthermore, gastrointestinal disorders often alter mucus properties, which can affect the pharmacokinetics of existing therapeutics.^9^ Knowledge of the production and characteristics of gastrointestinal mucus by goblet cells is therefore necessary for the design of targeted therapies, not only for conditions such as inflammatory bowel disease and other gastrointestinal disorders but ultimately also for all disorders treated by oral drugs.

Cell lineage allocation to form goblet cells in the human intestinal epithelium is modulated by the cell microenvironment during epithelial cell differentiation, including the concentration of growth factors,^10^ physical forces such as shear^11^ and the shape, angle, and cell position within the colon crypt,^12^ and chemical constituents formed by microbes and food metabolism.^13^ Thus, goblet cells respond to multiple cues to form a mucosal network designed to protect the intestinal epithelium. The continuous secretion of mucus by goblet cells not only blocks microbial access to the epithelial cells but also creates a brisk flow of mucus from the cell surface into the intestinal lumen and eventual excretion with fecal material or consumption by the microbiota.^14,15^ In vivo models using intravital microscopy have made possible the observation of intestinal mucus and its properties in living animals.^16^ By revealing the thickness, composition, and dynamic changes of the mucus layer in the intestinal tract, these models reveal the mucus layer in its natural state with the observation of dynamic changes in real time. While valuable, these animal models require complex experimental protocols, are not human-derived, and are not suitable for high-volume or throughput experimentation.^17–20^ *In vitro* models for the study of goblet cells and mucus have been created but these systems using primary cells are often short-lived, lack cell compartmentalization, possess no mucus layer, or require significant microdevice expertise.^21–27^ Microfluidic platforms with a monolayer of cells can display a thin mucus layer but these platforms typically comprise only differentiated cells with their attendant one-week lifespan.^25^ Organoids embedded in Matrigel possess 3D cryptlike protrusions and enable long-term culture due to the presence of stem cells, but lack an accessible mucus bilayer and open lumen, making bacteria coculture or luminal drug delivery challenging.^23^ Other systems utilize a 3D-shaped scaffold to support the formation of cryptlike structures complete with a stem/proliferative cell region, a differentiated cell zone, and a mucus layer.^26^ Remarkably these constructs can display the complex XYZ spatial patterning found *in vivo*. However, these platforms are typically relatively low-throughput in assay performance and possess high fabrication complexity and long maturation times.^26^ The many interfaces and 3D scaffolding also create microscopy imaging challenges due to light scattering or absorptive matrices. The complexity of these systems has largely limited their use to those skilled in both MPS development and primary intestinal cell culture.

To address these drawbacks, a planar model of intestinal crypts replete with self-renewing stem cell niches and differentiated cell zones was developed using both primary murine and human gastrointestinal stem cells.^28,29^ The platform was created using a flat, impermeable film patterned with an array of micrometer-scale through holes as a substrate held within a hanging basket. This substrate was then overlaid with a layer of collagen, and small or large intestinal stem cells plated on this array were cultured to confluence. Cells cultured above the through hole covered with collagen were supplied with the growth factors Wnt, R-spondin, and Noggin (WRN) supplied via the holes and diffusing through the collagen layer. This design enabled regularly spaced regions of stem/proliferative cell formation to create a stem cell niche. Since WRN was not supplied to cells beyond the through holes, spontaneous differentiation of the epithelial cell population occurred, forming differentiated cell zones around the stem cell niches. The luminal and basal regions of the tissue were accessible since the tissue was constructed at the base of a hanging basket. The 2D character of the tissue as well as the orderly array with known addresses for the stem cell niches supported facile imaging. The model thus enabled exquisite experimental control and study of the microenvironmental cues that impact stem cell turnover, cellular migration within the crypt, and cellular differentiation.^28–30^ Despite these many advantages, a physiological mucus layer was absent.

In the current study, we sought to create an intestinal MPS based on the above planar crypt system but possessing a high density of goblet cells and a thick mucus layer. Since goblet cell differentiation is partially governed by the concentration of WRN growth factors,^31^ the concentration profile of WRN surrounding the through holes was optimized for goblet cell formation by varying the thickness of the collagen layer to alter WRN diffusion both laterally and vertically within the collagen. The WRN radial concentration gradient was visualized by simulating WNT diffusion across the model device. The cells of the monolayer were characterized by immunofluorescence staining for the presence of goblet cells, colonocytes, enteroendocrine cells, and stem/proliferative cells. Goblet cell secretions were characterized for the binding of carbohydrate-selective proteins [*Lycopersicon esculentum* lectin (LEL) binding to *N*-acetylglucosamine [Gal-β (1→3)], *Lotus tetragonolobus* lectin (LTL) to L-fucose [Fucose α (1→2)], wheat germ agglutinin (WGA) to *N*-acetyl-d-glucosamine [GlcNAc] and N-acetyl-neuraminic acid (sialic acid)] and the presence of gel-forming mucins [mucin 5AC (Muc5AC), mucin 1 (Muc1), and mucin 2 (Muc2)] and secreted proteins [anterior gradient protein 2 homologue (AGR2), resistin-like molecule β (RELM-β), trefoil factor 3 (TFF3)]. Goblet cell formation and accumulation of a mucus layer were imaged by scanning electron microscopy (SEM) and assayed with immunofluorescence over varying culture times. This mucus layer was then characterized for thickness, resistance to bead penetration, and adherence to the underlying monolayer. The ability of the formed mucus layer to protect intestinal epithelial cells from toxin insults and microbial invasion was then evaluated by assessing β-actin and integrin β4 location in the presence of a *Staphylococcus aureus* toxin α-hemolysin or during coculture with the commensal bacterium *Lactobacillus rhamnosus* and the pathogen *Staphylococcus aureus*. This gut-mucus MPS will prove useful for the study of the relationship of epithelial cell renewal and differentiation, mucus dynamics, and microbiota–mucus–host interactions needed for enhancing our understanding of mucus physiology, pathophysiology, and drug interactions.

## 2 Materials and Methods

### 2.1 Expansion and Maintenance of Human Transversal Colon Cells

Human intestinal epithelial stem cells were isolated and expanded from the transverse colon of a cadaveric donor (RRID: 121 CVCL_ZR41; https://web.expasy.org/cellosaurus/CVCL_ZR41) as described previously.^32–34^ The donor was a 23-year-old male of unknown blood type. The stem cells were cultured on a flat 1 mm-thick slab of collagen in a stem cell medium (SM, Table S1). The expansion of these primary stem cells was performed after fewer than 8 passages for all of the experiments. Additional steps for expansion, maintenance, and passage of stem cells are described in the Supporting Information.

### 2.2 Bacterial Maintenance

The *Staphylococcus aureus* Newman strain was cultured under aerobic conditions in brain heart infusion media (BHI, MP Chemicals) at 37 °C. *S. aureus* was plated on BHI agar (with 15 g/L agar in a BHI medium), and a single colony was cultured for 18 h in a BHI medium for use in coculture experiments with epithelial cells. *Lactobacillus rhamnosus* GG (LGG, ATCC 53103, Microbiologics, Cat #01090P) was cultured in De Man, Rogosa, Sharpe (MRS) media (BD, Cat # 288130) at 37 °C. LGG was plated on an MRS agar plate (MRS with 15 g/L agar), and a single colony was cultured for 18 h in the MRS medium for coculture experiments.

### 2.3 Computational Modeling of Growth Factor Concentrations in Two-Dimensional Crypts

A finite element model of the concentration of a 40 kDa molecule was developed in COMSOL Multiphysics (version 5.5, COMSOL Inc., Burlington, MA) to simulate a 2D diffusion model of Wnt concentration (40 kDa). Other growth factors possess similar molecular weights (R-spondin-35 kDa, Noggin-26 kDa).^35–37^ In previous studies, the diffusion coefficient of fluorescein–dextran (40 kDa) was used to model WNT diffusion within an extracellular matrix^38^ due to the similar molecular weight of dextran to WNT. The Wnt concentration was simulated for two geometries of 10 through holes separated center-to-center by 175 or 350 μm. The geometric model consisted of vertically stacked rectangles, one representing each of the following: luminal medium, epithelial cell layer, collagen matrix, patterned epoxy film, and basal medium (Figure S3A). Domain-specific diffusion coefficients were assigned based on literature values (collagen hydrogel^39,40^ = 7 × 10^–12^ m^2^/s, and water^41^ = 1 × 10^–9^ m^2^/s). Growth factor diffusion through the 40 μm collagen-filled holes and a top layer of 10 μm collagen was modeled over time. Diffusion was simulated using the “Transport of Diluted Species” physics interface, which utilizes Fick’s second law of diffusion. Cellular uptake of WNT was incorporated by a reaction at the epithelial cell geometry with WNT consumption at a constant rate^42^ k= 4 × 10^–3^ s^–1^. Initial values were defined as 1 nM (∼30 ng/mL) for each growth factor in the basal media, while the luminal medium and collagen matrix were initialed at 0 nM. Boundary conditions were applied to the basal medium to represent a physiologically constant source of growth factors and to the luminal domain to represent an infinite sink. The diffusion across domains was assumed continuous; no additional barriers were imposed, and convective flow was not present. A time-dependent simulation was performed over 24 h with a 1 h step. Numerical accuracy was improved using a controlled mesh with an extremely fine element size. Spatial-temporal concentration profiles were evaluated to characterize growth factor diffusion in the collagen hydrogel.

### 2.4 Preparation of Epoxy Films to Support Planar Crypts

Epoxy films (40 μm-thick) possessing an array of 10 × 10 through holes were formed from 1002F50 ^43^ using photolithography as described previously.^28,29^ Each through hole was 70 μm in diameter, and the holes were spaced 175 or 350 μm (center-to-center). The epoxy film was attached to the bottom of a hanging basket (3401, Corning) with a medical-grade transfer tape (3M, 1504XL). The film/basket assembly was then treated with a plasma cleaner (Harrick Plasma Inc., model PDC-001) for 5 min. The film was chemically modified by exposure to 3-(aminopropyl)­triethoxysilane (APTES) vapor. After surface modification with amino groups, a 1% glutaraldehyde/water (v/v) solution (G6257-Sigma-Aldrich) was incubated with the assembly for 30 min at 20 °C. Glutaraldehyde reacted with exposed amine groups (added during APTES exposure) and displayed an additional unreacted aldehyde. A poly­(dimethylsiloxane) (PDMS) film was then placed in apposition to the epoxy film side of the assembly, i.e., the basal side of the hanging basket. Rat tail, type I collagen (150 μL, 1 mg/mL, pH 7.4) was then loaded into the luminal compartment of the hanging basket and gelled at 37 °C for 1 h. The exposed aldehydes of the chemically modified epoxy film were available to react with amino groups on the collagen, covalently cross-linking the collagen hydrogel to the epoxy film. The collagen hydrogel was then compacted by incubation at 40 °C for 20 h. When the PDMS film was detached from the backside of the epoxy film, the compacted collagen remained intact, spanning the surface of the through holes. The assembly was incubated in deionized water for 10 min. The assembly was then incubated in 75% ethanol in water for 5 min and air-dried. To determine the thickness of the collagen film, the collagen layer was incubated with fluorescein isothiocyanate (10 μg/mL in phosphate-buffered saline [PBS]) for 1 h. After the cells were rinsed, the thickness of the collagen layer was determined using a confocal laser scanning microscope. Just prior to use, the collagen-coated arrays were incubated with 1% Matrigel in PBS v/v at 37 °C for 12 h, followed by rinsing with PBS.

### 2.5 Generation of Stem/Proliferative Niches within Planar Crypts by Geometrically Constrained Diffusion of WRN

Colonic epithelial stem cells from 1 well (9.6 cm^2^ surface area) were harvested from their culture surface by incubation with 500 μL of TrypLE (12605028, Gibco) for 10 min at 37 °C. The expansion medium (EM, 5 mL, Table S1) was added to the culture, and the cells were suspended using a micropipette. The cell solution was centrifuged at 100*g* for 1 min. The cell pellet was resuspended in the EM (1.5 mL), and the cells (0.5 mL) were seeded onto the epoxy arrays by placement on the luminal side of the hanging basket assembly. The EM (1.5 mL) was added to the basal side of the hanging basket. Cells were then cultured at 37 °C with 5% CO_2_ for 3 days with (0.5/1.5 mL) EM luminal/EM basal reservoirs. After this time, the luminal EM was replaced with the DM (differentiation medium, 0.5 mL, Table S1), while the EM remained on the basal side. The luminal DM and basal EM were replaced every 24 h and the cells were further cultured for 5, 10, 15, or 20 days.

### 2.6 Mucus Thickness Analysis

Crypts polarized for 5, 10, 15, and 20 days were fixed with a Prefer fixative (Anatech Ltd., NC9053360) for 20 min at 22 °C, rinsed with 1× PBS, permeabilized with 0.5% Triton X-100 in PBS for 20 min at 22 °C, and stained with a 1:500 dilution of anti-Muc2 antibody (Alexa Fluor 488-conjugate, Jackson Immunoresearch, 115-545-003). The cells were also stained with Hoechst 33342 (1 μg/mL, 24 h, 22 °C). Red fluorescent beads (1 μm diameter, R0100, Thermo Fisher) were overlaid onto the fixed and stained tissue at a density of 10^8^ beads/mL. After a 15 min incubation, the beads settled onto the surface of the mucus. The epoxy film with attached collagen and fixed cells with the mucus layer was detached from the hanging basket and placed in an inverted position onto a glass slide. The location of the beads, immunostained mucin 2, and Hoechst 33342-stained cell nuclei were measured by confocal microscopy. The distance between the red fluorescent beads and nuclei (or the thickness of immunostained mucin 2) was used as a measure of the mucus layer thickness above the cells.

### 2.7 Addition of Bacteria or Toxins to Planar Crypt Arrays

As described above, human colonic epithelial cells were expanded for 3 days and polarized for 10 days on collagen-coated epoxy films (175 μm center-to-center holes). For experiments assessing the impact of *S. aureus* α-hemolysin, the luminal medium was replaced with phosphate-buffered saline (PBS, 0.5 mL) with 10 μg/mL *S. aureus* α-hemolysin (MedChem Express LLC, Cat# HY-P71825). The culture was then incubated at 37 °C for 24 h and assayed. For experiments assessing the impact of bacteria, bacteria were added to the luminal side of the epithelial cells. The luminal medium was replaced by a PBS buffer with *S. aureus* culture (0.5 mL, 1 × 10^3^ cells in total) or *L. rhamnosus* GG (LGG, 0.5 mL, 5 × 10^2^ cells in total). For experiments with *S. aureus*, the PBS buffer was supplemented with a 10% brain heart infusion (BHI) medium. For experiments with LGG culture, the PBS buffer was supplemented with 10% De Man, Rogosa, and Sharpe (MRS) broth. The cocultures were incubated at 37 °C for 24 h and then assayed. Samples without mucus were cultured with the DM containing 1.25 mM *N*-acetyl cysteine (NAC) for use as a control in experiments. β-Galactosidase (100 μg/mL) was added to the luminal compartment for 24 h prior to use in experiments.

Death of bacteria-exposed epithelial cells was quantified by measuring the area of the array positive for propidium iodide (PI, dead cells) in relation to the area positive for Hoechst 33342 (all cells).^44^ PI (2 μg/mL, Thermo Fisher, catalog no. P3566) and Hoechst 33342 (1 μg/mL, Thermo Fisher, cat. no. H1399) were incubated with cells for 24 h after epithelial cell exposure to bacteria. Fluorescence imaging of a subregion of the planar crypt array (1.1 cm^2^, 4 × 4 crypts) was performed using a 4× (0.16 N.A.) objective. Fluorescence excitation and emission wavelengths were as follows: Hoechst 33342 (Ex 405 nm, Em 430–470 nm) and PI (Ex 561 nm, Em 610–670 nm). Images were stitched to create a composite image of the planar crypt array. Image analysis was conducted using CellProfiler version 4.0, which identified and quantified the PI- or Hoechst 33342-positive area of the planar crypts using empirically set intensity thresholds. This protocol was adapted from a previously described method.^44^

### 2.8 Fluorescence Labeling of Proliferative and Differentiated Cells

To mark S-phase cells, the cells were cultured for 3 days in the EM and then polarized with the DM/EM luminal/basal medium for 4, 9, 14, or 19 days and then incubated with clickable EdU (10 μM) (Thermo Fisher Scientific, A10044) for 24 h at 37 °C; then, they were rinsed with PBS and incubated at 37 °C for 30 min with a red ALP substrate (Vector Laboratories, SK-5100) in Tris buffer (0.15 M Tris, pH 8.4). The cells were then fixed with a Prefer fixative (Anatech Ltd., 22 °C for 20 min), followed by permeabilization with 0.5% Triton X-100 in 1× PBS for 20 min. Clickable EdU that was incorporated into the cellular DNA was reacted with a Click-iT EdU Alexa Fluor 647 imaging kit (Thermo Fisher Scientific, C10340, 22 °C for 1 h). The tissue was then blocked with 3% bovine serum albumin (BSA) in PBS with 0.2% Triton X-100 and 0.05% Tween-20 for 1 h, at 22 °C. Primary antibodies for keratin 20 (Cell Signaling 130635 D9Z1Z, 1:400 dilution), chromogranin A (Proteintech, 10529-1-AP, 1:1000 dilution), OCLN (Proteintech, 13409-1-AP, 1:1000 dilution), and ZO-1 (Proteintech, 66452–1-Ig, 1:1000 dilution) were diluted in 200 μL of an immunofluorescence buffer (IF, Supporting Information) and incubated with the cells for 24 h at 4 °C. Then, the cells were washed ×3 times with IF buffer. A secondary antibody antirabbit IgG conjugated to Alexa Fluor 594 (Jackson ImmunoResearch, 111-585-003) was diluted to 1 μg/mL in 1% BSA in PBS with 1 μg/mL Hoechst 33342 and incubated with the cells for 1 h at 22 °C. After washing with PBS, the cells were imaged. Goblet cells and ZO-1 were immunolabeled using AF-488-conjugated antimucin 2 antibody (Santa Cruz, Sc-515032, 1:100 dilution). This antibody was diluted in 200 μL of IF with 1 μg/mL Hoechst 33342 and incubated for 24 h at 4 °C. Cells were washed ×3 times with IF and examined by a confocal laser scanning microscope (Olympus, Fluoview FV3000).

### 2.9 Fluorescence Labeling of Mucus Gel-Forming Proteins

Antibodies against Muc5AC (Invitrogen, MA3-1278, 1:200 dilution) and Muc2 (Santa Cruz Biotechnology, sc-7314, 1:200 dilution) were diluted in 200 μL of IF and incubated for 24 h at 4 °C. A secondary goat antimouse IgG conjugated to Alexa Fluor 488 (Jackson ImmunoResearch, 115-545-003) was diluted to 1 μg/mL in 1% BSA in PBS with 1 μg/mL Hoechst 33342 and incubated with the cells for 1 h at 22 °C. Other primary antibodies staining AGR2 (Invitrogen, PA5-34517, 1:200 dilution), RELM-β (Invitrogen, PA5-61896, 1:200 dilution), and TFF3 (Invitrogen, PA5-57279, 1:200 dilution) were detected by the secondary antibody goat antirabbit IgG conjugated to Alexa Fluor 594 (Jackson ImmunoResearch, 111-585-003) diluted to 1 μg/mL in 1% BSA in PBS with 1 μg/mL Hoechst 33342 and incubated with the cells for 1 h at 22 °C. Fluorescently labeled lectins, *Lycopersicon esculentum* lectin (LEL, Vector Lab, DL-1177, 1:100 dilution), *Lotus tetragonolobus* lectin (LTL, Vector Lab, FL-1321, 1:200 dilution), and wheat germ agglutinin (WGA, Invitrogen, W11261, 1:100 dilution) lectin were used to label carbohydrate groups on mucins. A labeled primary antibody against mucin 1 (Muc1, Invitrogen, sc-7313 AF594, 1:100 dilution) was diluted in IF with 1 μg/mL Hoechst 33342 and incubated with the cells for 24 h at 4 °C. After washing with PBS, the cells were examined by confocal microscopy.

### 2.10 Measurement of Cell Viability and β-actin and Integrin β4 Localization

To assess the viability of colonic epithelial cells after coculture with bacteria, the DM supplemented with 0.02 μg/mL Hoechst 33342 was added into both the luminal (0.5 mL) and basal (1.5 mL) compartments and then incubated at 37 °C for 1 h. After washing with 1× PBS, 2 μg/mL propidium iodide (PI, Thermo Fisher, catalog no. P3566) in PBS buffer (1.5 mL) was added into the basal compartment, followed by a 20 min incubation at 37 °C. Hoechst 33342 labeled all epithelial cells, while PI stained only dead epithelial cells. Primary antibodies against β-actin (Santa Cruz Biotechnology, sc-81178, 1:200 dilution) and integrin β4 (Santa Cruz Biotechnology, sc-9090, 1:100 dilution) were diluted in 200 μL of IF and incubated for 24 h at 4 °C. Goat antimouse IgG conjugated to Alexa Fluor 488 (Jackson ImmunoResearch, 115-545-003) was used to detect β-actin, and goat antirabbit IgG conjugated to Alexa Fluor 594 (Jackson ImmunoResearch, 111-585-003) was used to detect integrin-β4. The two secondary antibodies were mixed together at 1 μg/mL for each antibody in 200 μL of 1% BSA in PBS with 1 μg/mL Hoechst 33342 and incubated with the cells for 1 h at 22 °C. The cells were washed ×3 times with PBS and examined by confocal microscopy.

### 2.11 Fluorescence Confocal Microscopy

A confocal laser scanning microscope (Olympus, Fluoview FV3000, Waltham, MA) equipped with 405, 488, 561, and 640 nm laser diodes in conjunction with a galvanometer scanner was used to acquire all fluorescence data. Laser-based excitation and emission wavelengths (peak values) for the different fluorophores were Hoechst 33342:430/470 nm; Cy5:650/750 nm; Alexa Fluor 488:505/545 nm; and Texas Red: 600/640 nm. A 4× objective (N.A. 0.16 UplanSApo4X), regions of interest (ROIs) with a 1× digital zoom (2.21 μm/pixel, 1024 pixel2 area), was used to collect at least 3 Z-series optical sections collected at a step size of 10 μm to account for the sample thickness. A 10× objective (N.A. 0.4 UPLSAPO 10 × 2) with 0.5 magnification was used for imaging (0.4023 pixels/micron resolution, and 150 μm depth for each of 10 Z-images or 210 μm depth for each of 30 Z-images). For higher-resolution images, a 30× oil immersion objective (N.A. 1.05, UPlanSApo30XS) with 1× digital zoom was employed.

### 2.12 Scanning Electron Microscopy

Fixed, unstained arrays with and without mucus were processed with 3 washes with DI water followed by consecutive incubations (200 μL for 10 min at 22 °C) in 50, 70, 80, 90, and 100%, ethanol mixed into water. Dehydrated cells were then incubated with 200 μL of ethanol and hexamethyldisilazane (HDMS) at 3:1 for 15 min, 2:1 for 30 min, 1:1 for 30 min, and 100% HDMS until evaporation (24 h). Samples were coated with gold at 10 mA and 7 nm/min in an EMS Quorum 150T ES sputter coater at a density of 19.32 g/m^3^. A scanning electron microscope (SEM) (Phenom Pro, Thermo Fisher) was used for image acquisition operating at 10 kV.

### 2.13 Image Analysis

Image analysis was performed using Imaris X64 v9.8.2 (imaris.oxinst.com, Oxford instruments) or the open-source image processing software ImageJ/Fiji version 2.14.0/1.54f (http://imagej.net).^45^ Fiji was used to identify the image area above an empirically set threshold for each of the fluorophores, and the image area positive for the marker was calculated. The image regions comprised 10 × 10 planar crypt units. This region was either 1750 or 3500 μm on a side for arrays with a 175 or 350 μm center-to-center spacing for through holes, respectively. The percentage coverage of each stain (EdU, Hoechst 33342, PI, or antibody-based labels) was calculated as the cell area positive for the fluorescent stain divided by the area within a region of interest (ROI) and multiplied by 100 (coverage %). Cell viability on the arrays was represented by the PI-positive area divided by the Hoechst 33342-positive area. Cross-sectional images were created with Fiji by stacking Z slices into two-dimensional representations by using maximum intensity z-projections for mucus thickness measurements. The fluorescence intensity of β-actin and integrin β4 immunostaining was quantified by measuring the fluorescence intensity along the luminal or basolateral cell aspects, respectively, and normalizing the range from 0 to 1. Stem/proliferative cell niche zone diameters were measured using 50 different crypts per condition. All data utilized three biological replicates for each condition.

### 2.14 Statistical Analysis

Data are presented as mean ± standard deviation (SD). Differences between means from multiple comparisons were determined using 2-way ANOVA. Differences between means from separate groups were calculated using an ordinary one-way ANOVA. Tukey’s honestly significant difference post hoc test was conducted. The level of significance was indicated by the calculated P value. Asterisks in figures are defined as follows: * *P* < 0.05; ** *P* < 0.01; *** *P* < 0.001; “ns” not significant *P* > 0.05. Statistical analysis and graphical illustrations were performed using GraphPad Prism 10 (version 10.4.1 for Windows 64-bit, GraphPad software, Boston, MA, www.graphpad.com).

### 2.15 Ethics Statement

The studies involving humans were approved by the procurement organization. All of the methods were carried out in accordance with relevant guidelines and regulations stipulated by the National Institutes of Health. The experimental protocols were approved by the University of Washington. The cell line used in this work was derived from deidentified cadaveric transplant donor intestines, made available through an NIH-funded biobank. The original tissues used to create this biobank were obtained from a federally designated organ procurement organization. Transplant donors were consented and deidentified by the procurement organization. The studies were conducted in accordance with local legislation and institutional requirements. The human samples used in this study were acquired from cadaveric donors. Written informed consent for participation was not required from the participants or the participants’ legal guardians/next of kin in accordance with the national legislation and institutional requirements.

## 3 Results

### 3.1 Design Overview

The objective of this study was to create an intestinal cell platform that was easy to use and was composed of a long-term planar crypt array with a stem cell niche and a differentiated cell zone plus that demonstrated a sufficiently thick mucus layer to support bacterial coculture (Figure 1).^28,29^ Briefly, an epoxy film with a 10 × 10 array of through holes attached to the base of a hanging basket (Figure 1A,B) was coated with a thin collagen layer to provide a biomimetic substrate for the culture of primary intestinal epithelial cells and which prevented cell migration to the basal side of the epoxy film (Figure 1C). Stem/proliferative cells were cultured on the collagen scaffold under a medium (the EM medium containing the WRN growth factors) that encouraged proliferation and discouraged differentiation (Figure 1D). Once the cells formed a confluent monolayer, the luminal medium was replaced with a medium without WRN (DM medium; Figure 1E,F). The cells above the through holes formed a localized stem/proliferative cell microniche by virtue of the continual exposure to WRN (Figure 1G–I). Cells at a distance from the microholes received no WRN and differentiated, but with an insufficient number of goblet cells to form a mucus layer (Figure 1G,H). Prior work demonstrated that the vasoactive intestinal peptide (VIP) promoted goblet cell formation in a primary epithelial monolayer.^21^ Since vasoactive intestinal peptide receptor 1 (VPAC1) is present predominantly on the luminal surface of colonic epithelial cells,^46^ the VIP was added into the luminal medium at the time of differentiation. After 5 days, goblet cells were detected around the differentiated zone under these conditions; however, a mucus layer did not form above the patterned intestinal epithelium (Figure S2). To generate sufficient goblet cells to form a mucus layer, the planar crypt array required additional optimization.

**Figure 1:**
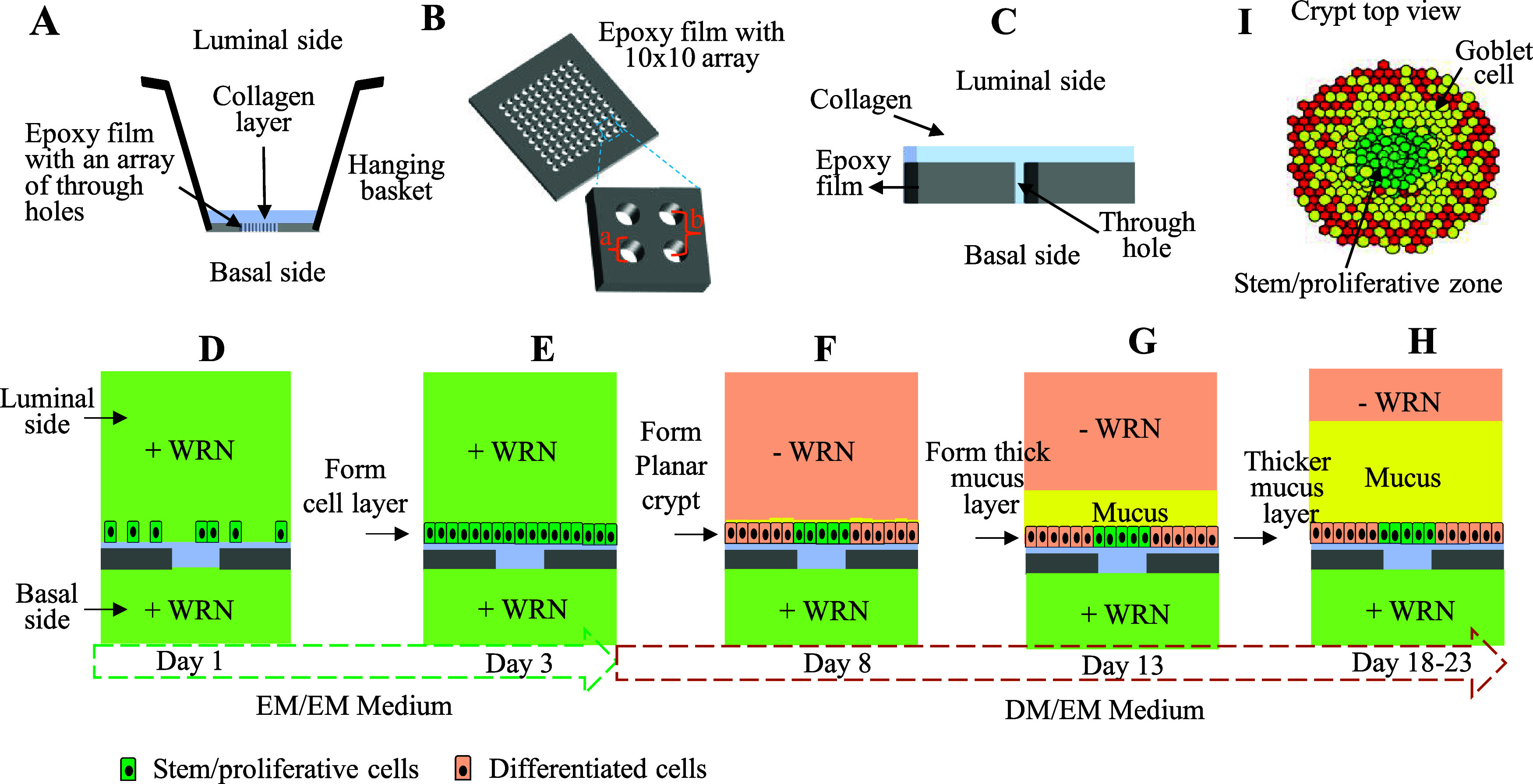
Schematic of planar crypt arrays. (A) Side view of a hanging basket with key components marked. (B) Tilted side view of an array of 10 × 10 through holes (a = 70 μm in diameter) with a center-to-center gap of 175 μm (labeled as b) in the photoresist epoxy film (40 μm-thick). (C) Cutaway of a single through hole in the epoxy film with an overlying collagen layer. Steps in the creation of a polarized planar crypt: (D) on day 1, cells from cultures comprised predominantly of stem/proliferative cells are placed onto the planar crypt arrays; (E) by day 3, a confluent monolayer has formed above a thin collagen layer covering the array surface, and WRN is then removed from the luminal compartment; (F) on day 8 of total culture (3 days in the expansion EM/EM and 5 days in the polarization DM/EM), the planar crypts have formed with stem/proliferative cells localized in the array regions above the through holes; (G) by 13 days, a mucus layer has begun to form above the crypt arrays; and (H) by 18–23 days, a thick mucus layer covers the planar crypt array. (I) Top view schematic of a single fully formed planar crypt (red = colonocytes, yellow = goblet cells, green = stem/proliferative cells).

### 3.2 Optimization of Collagen Thickness

Lineage allocation toward secretory cells and ultimately goblet cells is partially governed by the concentration of the growth factors WRN. The concentration profile of WRN above the through holes in turn depends on the thickness of the collagen since WRN will diffuse both laterally and vertically within the collagen once transiting the hole in the epoxy film. To optimize the collagen thickness, varying volumes (120 to 200 μL) of collagen were used to form the compacted collagen layer above the epoxy film. The compacted collagen layer thickness was measured by confocal microscopy after reaction with fluorescein isothiocyanate and ranged from 5 to 19 μm (Figure S1). Primary human colonic epithelial cells were cultured above the various collagen layers for 3 days in a medium with growth factors (the EM in the luminal and basal reservoirs) to permit the cells to proliferate across the collagen layer. The luminal medium was then replaced with the DM, while the basal reservoir medium contained the EM to initiate polarization of the array and formation of distinct cell zones. After 9 days under these DM/EM conditions, the cells were pulsed for 24 h with EdU to identify S-phase cells and then stained with Hoechst 33342 to identify all cells (Figure S1B–D). On the thicker collagen layers (13–19 μm), Hoechst 33342 staining covered nearly the entire array, suggesting that a confluent monolayer covered the surface (Figure S1B,E). However, EdU-positive cells were also distributed throughout the surface and a discrete stem/proliferative cell zone failed to form (Figure S1B,D). This was most likely due to the extensive lateral diffusion of WRN through the thick collagen prior to reaching the cell monolayer.

Thinner collagen films (5–10 μm) displayed confined EdU+ cell zones centered above the through holes (Figure S1B). The 5 and 10 μm-thick films also exhibited extensive cell coverage as a high percentage of the area was positive for Hoechst 33342. While the area positive for Hoechst 33342 was not significantly different between the two collagen thicknesses, the 5 μm-thick film possessed occasional cell-free zones. Thus, only the 10 μm-thick collagen film supported both a discrete stem/proliferative cell zone as well as a fully contiguous cell monolayer across the entire array and, therefore, was used for all subsequent studies. Goblet cells (identified by Muc2+) were formed on the crypt arrays with a 10 μm-thick collagen film (Figure S2, 350 μm center-to-center hole spacing); however, a contiguous mucus layer was not present. *In* *vivo*, lineage allocation toward the goblet cells occurs as growth factor signals are attenuated but not eliminated as the cells move along the crypt axis.^47^ Thus, it was possible that the large distance between the through holes or stem cell zones resulted in the cells experiencing a suboptimal growth factor signal to fully promote goblet cell formation, as the cells rapidly moved out to the periphery of the crypt units.

### 3.3 Optimization of the Distance between the Through Holes

The distance between the through holes in the epoxy film determines the WRN radial concentration gradient. In turn, the duration of cell exposure to WRN influences cell lineage allocation and migration from the cell division location above the through holes. The distance between the through holes in the epoxy film was varied (a center-to-center gap of 175 or 350 μm), with identical diameters for both through hole gaps (Figure 2A). The cultures were grown in the EM/EM for 3 days (day 1–3) and then for a further 5 to 20 days in the DM/EM (total days in cultures: 8, 13, 18, and 23) (Figure 2B). One day prior to assay, the cells were pulsed with EdU to mark the S-phase or proliferative cells on the arrays (Figure 2C). By day 8 of culture, both arrays possessed stem/proliferative cell zones which were not significantly different in size (151 ± 16 μm and 154 ± 13 μm diameters for 350 and 175 μm gaps, respectively, *p* = 0.28, *n* = 50, Figure 2D,E). Over time, the stem/proliferative cell zones decreased significantly in size for both arrays relative to the day 8 size (86 ± 23 μm and 117 ± 12 μm diameters for 350 and 175 μm gaps, respectively, on day 23, *p* = 0.0351, *n* = 50, Figure 2D,E). Not surprisingly, when viewed as a percentage of the total array area, the EdU+ area was significantly lower for the larger compared to the smaller gap arrays (Figure 2F,G).

**Figure 2:**
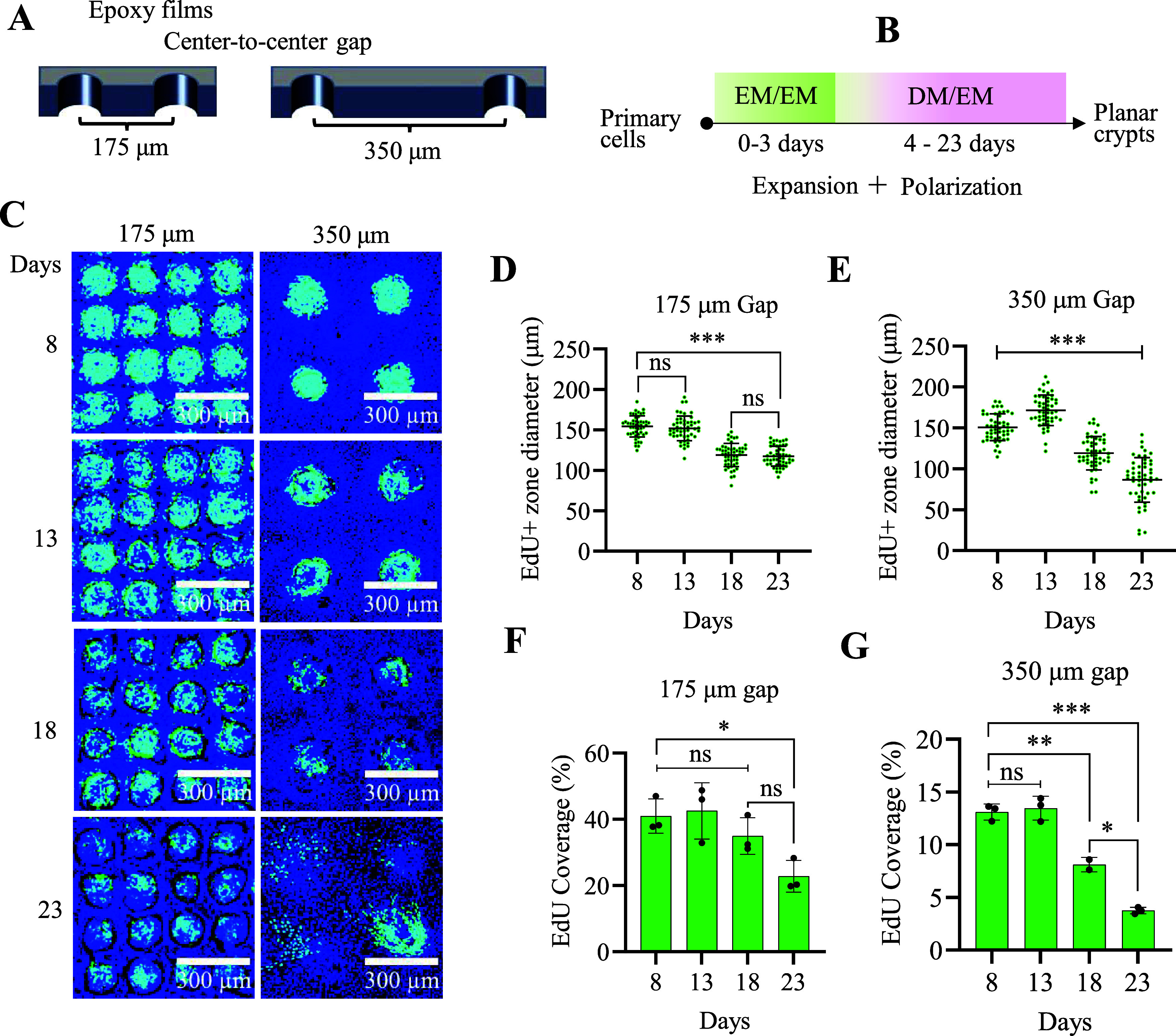
Impact of the distance between the through holes on the formation of polarized planar crypts. (A) Schematic of the 2 tested distances between through holes. (B) Schematic of cell culture steps and timing to form planar crypts in these assays. (C) Formation of the stem/proliferative zones over time for the array geometries. EdU (green); Hoechst 33342 (blue). (D, E) Size of the EdU+ zone over time for the two array geometries. *n* = 50 crypts were analyzed for each day (8, 13, 18, and 23) for each gap (175 and 350 μm). One-way ANOVA multiple comparisons by Tukey. ***p < 0.0001; ns for *P* value >0.05. Percent positive area for EdU; (F) 175 μm gap or (G) 350 μm gap. *N* = 3 biological replicates were imaged, i.e., three arrays, and *n* = 16 crypts were analyzed for each of the three biological replicates. One-way ANOVA multiple comparisons by Tukey. ***p < 0.0001; **p < 0.001; *p < 0.05, ns for *P* value >0.05.

The formation of a differentiated cell zone on the differently sized arrays was examined by assaying for four differentiated cell markers (ALP for absorptive colonocytes, Muc2 for goblet cells, ChgA for enteroendocrine cells, and KRT20 for all differentiated cell types) (Figure 3). The cultures were grown in the EM/EM for days 1–3 and in the DM/EM for 10 days (Figure 3A). The percentage area positive for EdU was significantly greater for the 175 μm gap arrays compared to the 350 μm gap arrays. This was most likely due to the identical through hole size yet much smaller distance between through holes on the 175 μm gap compared to the 350 μm gap arrays. The percentage area positive for KRT20, covered by Hoechst 33422, or positive for ALP was not significantly different for the larger 350 μm gap compared to the 175 μm gap (Figure 3B–D). Proteins (ZO-1 and OCLN) associated with tight junctions were immunostained on both 350 and 175 μm gap arrays, and the percentage area positive for the immunostain was not significantly different for the two-sized arrays. Surprisingly, enteroendocrine cells were significantly greater in number on the smaller 175 μm gap array relative to that on the larger gap array on day 10 (Figure 3B–D). Similarly, the percentage area positive for Muc2 was greater for the 175 μm gap vs the 350 μm gap array, suggesting that the growth factor profile on the smaller gap array supported secretory cell formation relative to the large gap array. The location of the majority of the Muc2+ area either overlapped the stem/proliferative cell zone or was located adjacent to this zone with many Muc2+ cells at a distance from the through holes. The higher overall density of through holes on the small gap arrays, therefore, supported a greater percentage of the Muc2+ surface area relative to the large gap arrays. These results are consistent with the *in vivo* cell locations with absorptive colonocytes localized to the luminal intestinal surface but not present within the crypts. In contrast, goblet cells are found at a high density within the crypts, as well as along the luminal-facing epithelium. The high Muc2+ surface area on the small gap arrays suggested that these arrays possessed a greater density of goblet cells compared with the large gap arrays.

**Figure 3:**
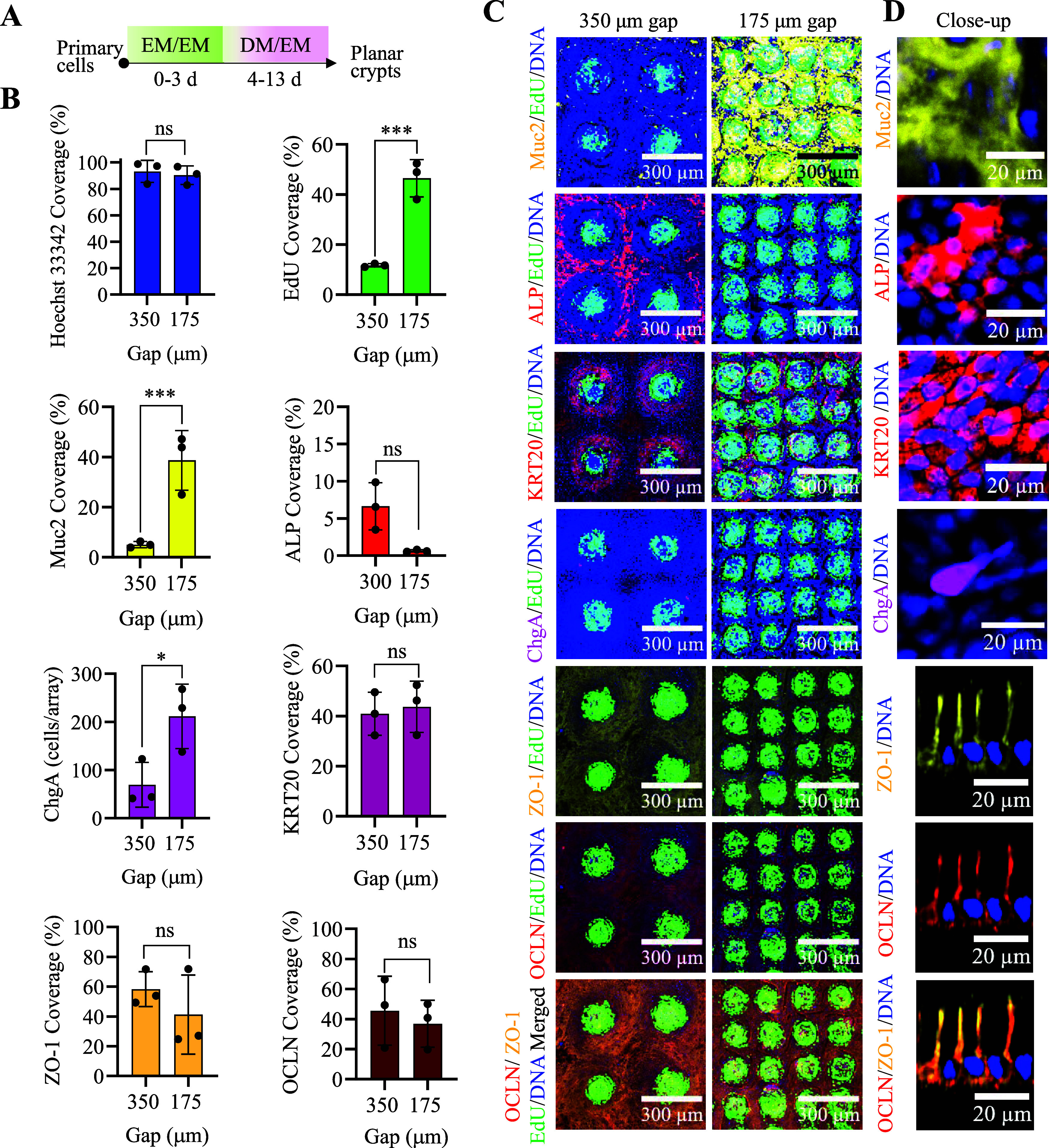
Impact of the distance between the through holes on the formation of differentiated cells. (A) Schematic of cell culture steps and timing to form planar crypts in these assays. (B) Quantification of Muc2, ALP, KRT20, ChgA, EdU, ZO-1, OCLN, and Hoechst 33342 fluorescence staining for the 2 different array geometries. The percentage coverage of stains was calculated as the array area positive for the fluorescent stain (above an empirically set threshold) divided by the total area of the region examined. (C) Fluorescence microscopy images of day 13 planar crypt arrays stained for EdU incorporation (green), Muc2, (yellow), ALP (red), KRT20 (red), ChgA (magenta), ZO-1 (yellow), and OCLN (red). Images of 4 or 16 planar crypts are shown for the 350 and 175 μm gap arrays, respectively. *N* = 3 biological replicates were imaged, i.e., three arrays, and *n* = 16 crypts were analyzed for each of the three biological replicates. (D) Close-up view of fluorescent stains for Muc2, ALP, KRT20, ChgA, ZO-1, and OCLN. The images with ZO-1 and OCLN are *X*–*Z* slices, while the others are *X*–*Y* slices. All samples were stained with Hoechst 33342 (blue). Two-way ANOVA multiple comparisons by Tukey. ***p < 0.0001; *p < 0.05, ns for *P* value >0.05. ChgA was analyzed as *n* = 100 crypts for each biological replicate and each array geometry.

The concentration of WNT on the arrays with the different sized gaps (a 10 μm-thick collagen film) was computationally modeled to visualize the concentration gradient differences between the two array types (Figure S3A,B). The WNT concentration map in the *XZ* dimension for 350 μm gap arrays suggested that [WNT] dropped to zero by 175 μm from the center of a through hole so that most cells between the through holes experienced very little exposure to WNT (Figure S3C). In contrast, for the 175 μm gap arrays, the WNT concentration never dipped to less than ∼40% of that directly above the through holes. Thus, on the smaller gap arrays, the cells were always exposed to WNT, although at different concentrations in the periphery relative to the crypt center. These simulations suggest that the different geometry of the arrays results in very different WNT exposure profiles for cells between the center hole and the intercrypt regions and are consistent with prior experimental data demonstrating that secretory cell formation requires a reduced (but nonzero) [WNT] to form.^48^ Based on the experimental data and simulations, 175 μm gap arrays were used in all subsequent experiments for optimal goblet cell formation.

### 3.4 Goblet Cell Characterization

Goblet cells formed on the arrays were characterized for the presence of mucins (Muc5AC, Muc1, Muc2), an antimicrobial peptide (RELM-β), and mucin maturation proteins (AGR2, TFF3) (Figure 4A).^49,50^ As expected, a significantly (*p* = 0.023) greater area of the culture surface was covered by Muc2 than by Muc1 and Muc5AC recapitulating *in vivo* physiology (Figure 4B–D). TFF3, RELM-β, and AGR2 were all present within the mucus, suggesting that the mucus might be competent as a barrier (Figure 4B–D).

**Figure 4:**
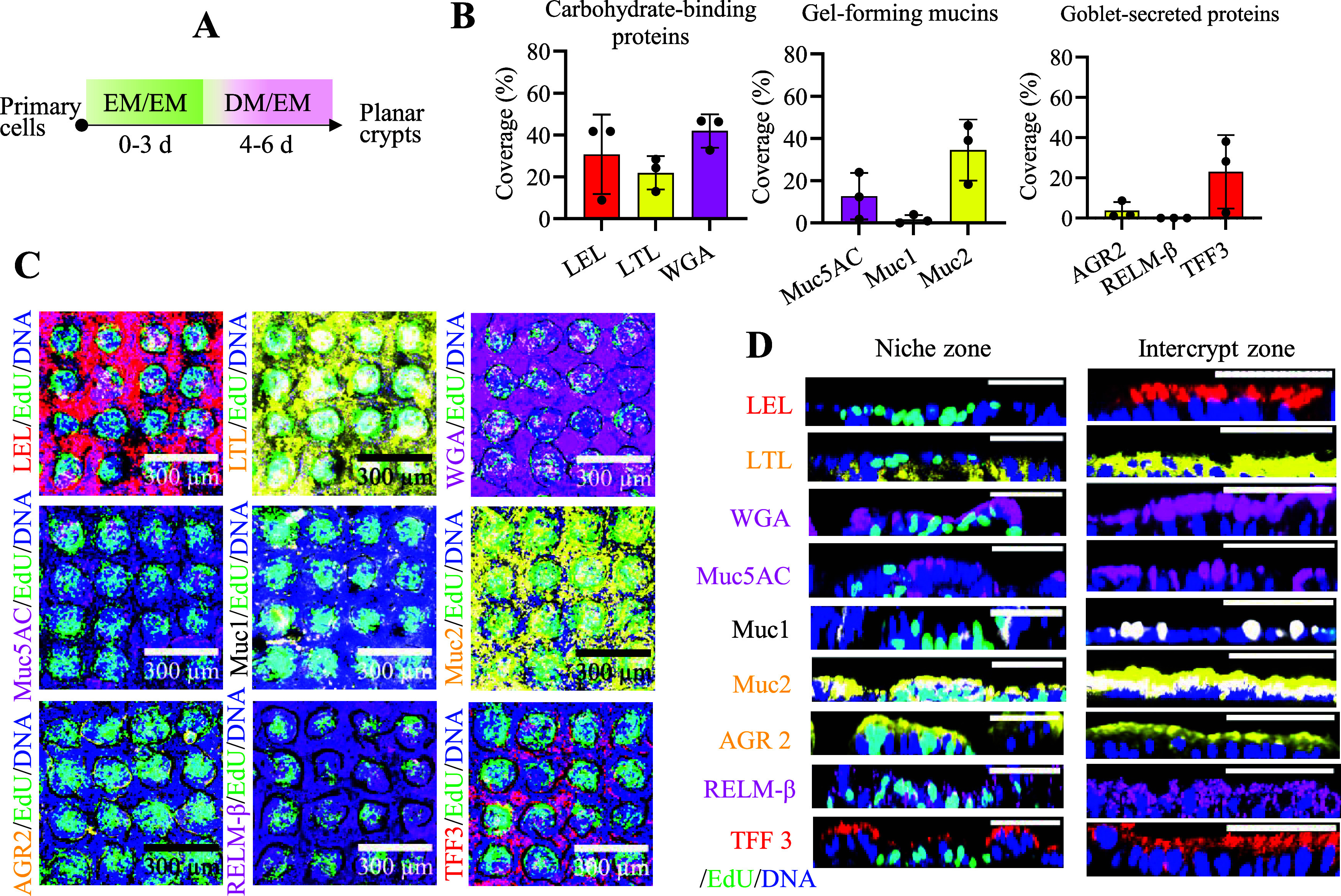
Goblet cell characterization. (A) Schematic of cell culture steps and timing to form the planar crypts. (B) Percentage area of the array positive for fluorescent lectin binding (LEL, LTL, WGA), immunostained mucins (Muc5AC, Muc1, and Muc2), and immunostained goblet cell proteins/peptides (AGR2, RELM-β, and TFF3). Each symbol represents a single data point and the average with a standard deviation is plotted. (C) Fluorescence images (*XY* plane) of 9 planar crypt arrays (16 polarized crypts, 4 × 4 array) stained with different goblet cell markers. (D) Fluorescence images (*XZ* plane) showing a close-up of a single stem/proliferative niche (each image in the left 9 panels) and the corresponding intercrypt zones (right 9 panels); scale bar = 100 μm. In all images, *N* = 3 biological replicates were imaged, i.e., three arrays, and *n* = 16 crypts were analyzed for each of the three biological replicates. Muc2, LTL, and AGR2 (yellow); LEL and TFF3 (red); WGA, Muc5AC, and RELM-β (magenta); Muc1 (white); Hoechst 33342 (blue); EdU (green).

Lectin-based binding (LEL, LTL, WGA) has been used to assess goblet cell heterogeneity based on the differential display of carbohydrates on the cell’s mucins.^12,51^ The lectins LTL and WGA localized to goblet cells at the stem/proliferative niche as well as in the differentiated cell zones (Figure 4B–D). However, LEL was observed primarily in the differentiated or interniche zone. These results mimic those observed *in vivo* as well as in a 3D intestine-on-chip MPS (Figure 4B–D).^12,26^ Taken together, this simple 2D crypt array displayed attributes of functional mucus and goblet cells including the heterogeneity of goblet cells based on their location within the crypt.

### 3.5 Impact of Culture Time on Muc2 Accumulation

To characterize Muc2 accumulation over time, the culture time in the DM/EM was extended to 20 days (Figure 5A). The percentage surface area positive for Muc2 significantly increased between days 8 and 23 whereas the percentage of Hoechst 33342+ did not significantly change over this time (Figure 5B,C). These data suggest that the total cell density was constant during this time, but either more Muc2+ was produced by each goblet cell or there were more goblet cells. During the 23 day culture time, a layer of Muc2 began to span the array, even flowing into device regions without an underlying crypt array (Figure 5D,H). These data suggested that over the culture from day 8 to 23, a mucus layer was formed covering the crypt array.

**Figure 5:**
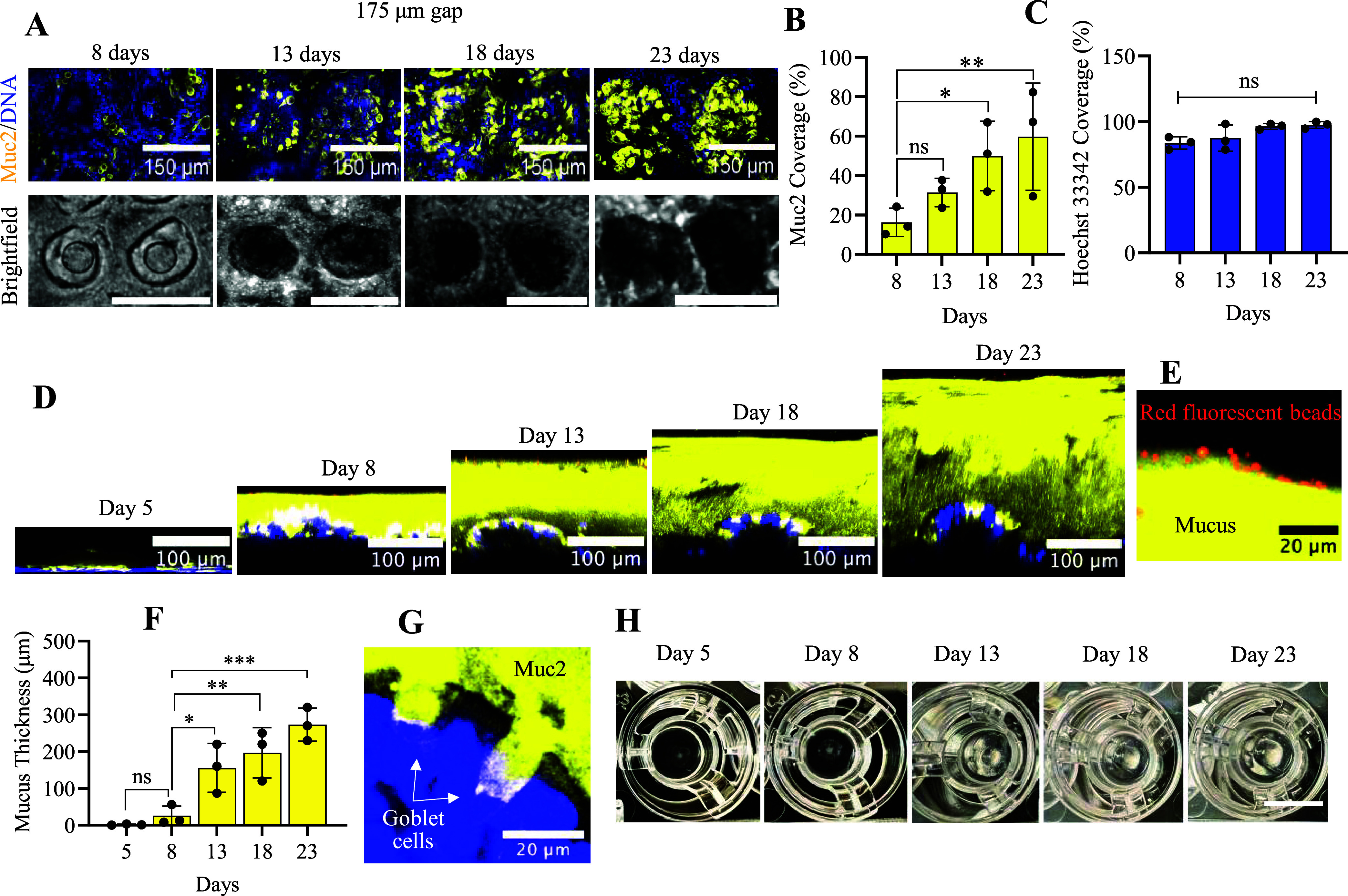
Enrichment of goblet cells and formation of a mucus layer. (A) Fluorescence (top row) and brightfield (bottom row, scale bar = 200 μm) images (*XY* plane) of 2 planar crypts at different days of culture. (B, C) Percentage area of the array staining positive for Muc2 or Hoechst 33342 plotted against the time of culture. (D) Cross-sections (*XZ*) of the mucus layer above a single planar crypt on an array at different days of culture. (E) Close-up of red fluorescent beads (1 μm) resting on the mucus layer. (F) Mucus thickness above the plane of the epithelium was plotted against time. (G) Goblet cells in the process of MUC2 exocytosis. (H) Photographic images of mucus accumulation over time on the culture surface; scale bar = 12 mm. In all images, Muc2+ (yellow); Hoechst 33342 (blue); and red beads (red). *n* = 3 technical replicates for each condition were analyzed. Two-way ANOVA multiple comparisons by Tukey. ***p < 0.0001; **p < 0.001; *p < 0.05, ns for *P* value >0.05.

### 3.6 Characterization of the Mucus Layer above the Crypt Array

To quantify the thickness of the mucus layer, the distance between red fluorescent beads (1 μm diameter) and the Hoechst 33342-stained epithelial cells was measured over time for Muc2-immunostained cultures. After addition, the beads settled onto the top surface of the Muc2+ area above the epithelial cells (Figure 5D,E). A significant increase in the height of the red beads above the cell nuclei (Hoechst 33342+) occurred on each measurement day between days 5 and 23 (Figure 5D,F). During the 23 day culture time, the underlying epithelial cells became folded, developing a wrinkled texture but with preservation of the stem/proliferative and differentiated cell zones (Figure S4). Goblet cells (length = 25 ± 3 μm, width = 13 ± 8 μm, n = 25) in the process of MUC2 exocytosis were readily visualized within the epithelial cell monolayer (Figure 5G). The mucus over time was increasingly difficult to dislodge from the culture surface, as it remained attached to the culture after extensive processing (fixation, permeabilization, rinses, and submerged incubations overnight, Figure 5H). These data suggested the formation of a dense layer of mucus tightly attached to the cells.

### 3.7 Protection of Epithelial Cells from Toxins by Mucus

Since one role of the colonic mucus is to protect the epithelial cells from toxins, the ability of the mucus layer to shield the epithelial cells from α-hemolysin was assessed (Figure 6A,B). α-Hemolysin is secreted by *S. aureus*, a frequent pathogen of the colon, and binds to the ADAM10 (desintegrin and metalloproteinase domain-containing protein 10) protein on the host epithelium through a protein–protein interaction.^52,53^ This toxin forms pores in the host cell membrane admitting calcium followed by calcium-mediated reactions disrupting the cytoskeleton and signaling proteins and creating an osmotic imbalance across the cell membrane. Planar crypt arrays were formed over 10 days, accumulating a 100 μm-thick mucus layer (Figure S5). Planar arrays lacking mucus were prepared by adding NAC to the luminal medium. NAC acts as a mucolytic agent by reducing disulfide bonds in mucin glycoproteins through a thiol–disulfide exchange reaction.^54,55^ One day prior to adding the toxin, the tissues were also pretreated by adding β-galactosidase to the luminal media to break down complex sugars in any remaining mucins. The presence or absence of the mucus layers was confirmed with SEM microscopy of the stem/proliferative and intercrypt zones (Figure S5). α-Hemolysin was added to the luminal medium for 1 day, and then cells were fixed and immunostained for β-actin and integrin-β4 as well as stained with Hoechst 33342. Epithelial cells with mucus (with or without toxin) and epithelial cells without mucus and without toxins displayed a physiologic localization of immunostained integrin-β4 and β-actin on the basolateral or luminal side of each cell, respectively (Figure 6C,E).^56^ When a reconstructed Z section (*XZ* plane) was used to visualize the cells, these samples possessed β-actin staining along the cell upper border, as expected for a contiguous, undisrupted cell monolayer (Figure 6E). In contrast, the sample without mucus but with added α-hemolysin displayed a loss of actin from the luminal cell edges, suggesting cytoskeletal disruption in the cells (Figure 6C,E). In this sample, all zones (the stem/proliferated region and differentiated zone) of the planar crypts were impacted by the toxin. This sample also displayed regions without apparent β-actin immunostaining when viewed in the *XZ* plane. Integrin-β4 immunostaining was also altered on the basolateral side of cells exposed to α-hemolysin without mucus relative to the other samples (Figure 6D,E). These findings suggested that the mucus served as a protective barrier, maintaining epithelial integrity against α-hemolysin (33 kDa) or at least delaying its diffusion to the epithelium within the assay time frame.

**Figure 6:**
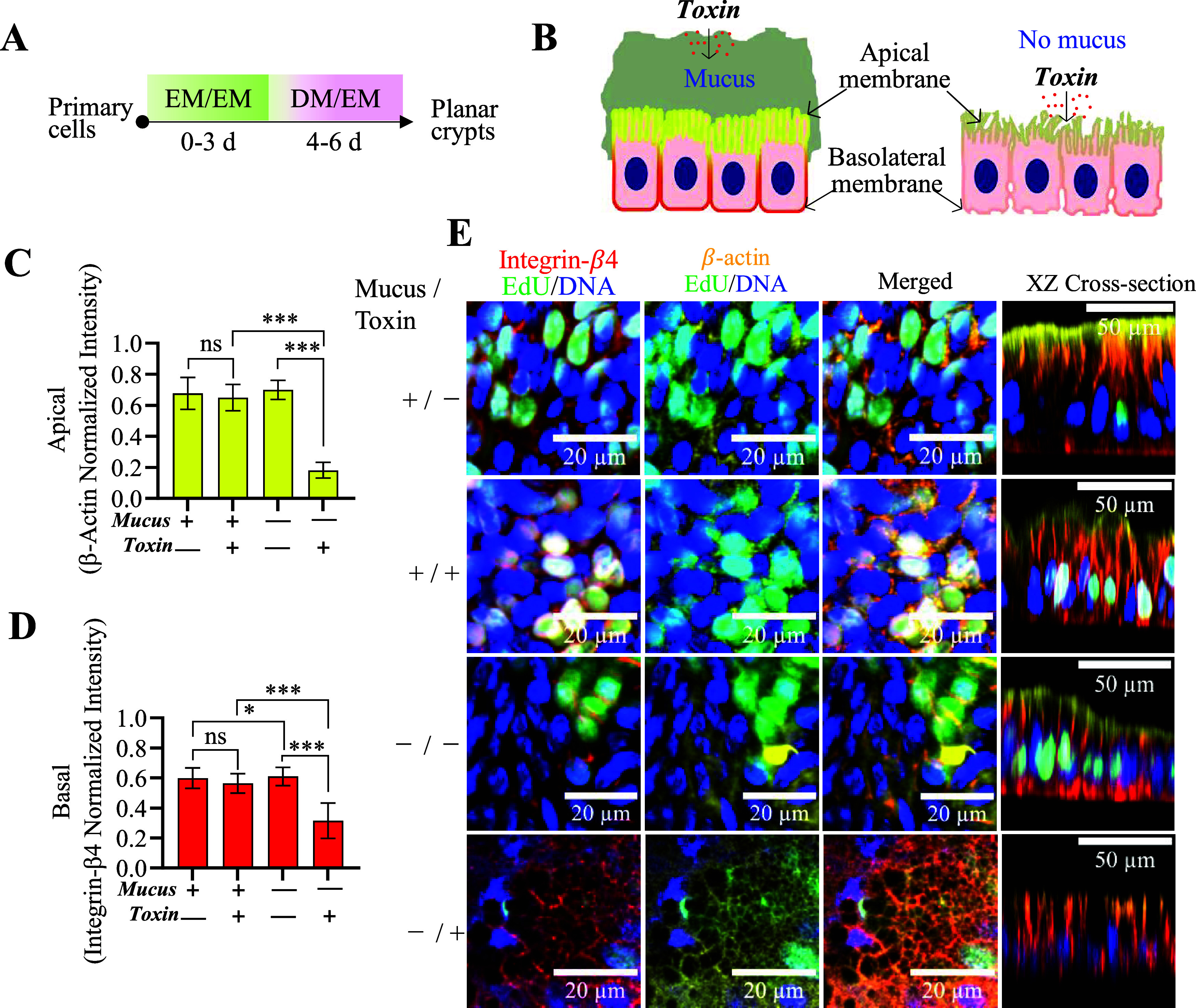
Impact of α-hemolysin on planar crypts with and without a mucus layer. (A) Schematic of cell culture steps and timing to form planar crypts in these assays. (B) Schematic of the α-hemolysin toxin effect on the luminal and basal cell aspects with and without mucus protection. (C, D) Normalized fluorescence intensity of immunostained β-actin and integrin-β4 for 4 different conditions: mucus without toxin, mucus with toxin, no mucus and no toxin, and no mucus with toxin. (E) Fluorescence images of cultures with and without mucus and with and without added α-hemolysin are shown (*XY* scale bar = 20 μm or *XZ* scale bar = 50 μm). β-actin (yellow), integrin-β4 (red), EdU (green), and Hoechst 33342 (blue). *N* = 3 biological replicates were imaged and analyzed. Two-way ANOVA multiple comparisons by Tukey. ***p < 0.0001, *p < 0.05, ns for *P* value >0.05.

### 3.8 Protection of Epithelial Cells from Bacteria by Mucus

*L. rhamnosus* is a Gram-positive commensal facultative anaerobe often used as a probiotic to increase the health of the colon. *L. rhamnosus* was added to the luminal reservoir of planar crypts with and without a mucus layer. The planar crypts were incubated for 1 day, and epithelial cell viability was measured by staining with propidium iodide to mark dead cells and Hoechst 33342 to label all cells. Control samples (mucus with bacteria, no mucus and no bacteria, and mucus without bacteria) possessed very few dead cells and were not significantly different in their number of dead cells (Figure 7). In contrast, epithelial cells without a mucus layer and *L. rhamnosus* possessed significantly more dead cells than did the control samples. These data suggest that the mucus layer is required for epithelial cell protection even in the presence of a commensal bacterium such as *L. rhamnosus*.

**Figure 7:**
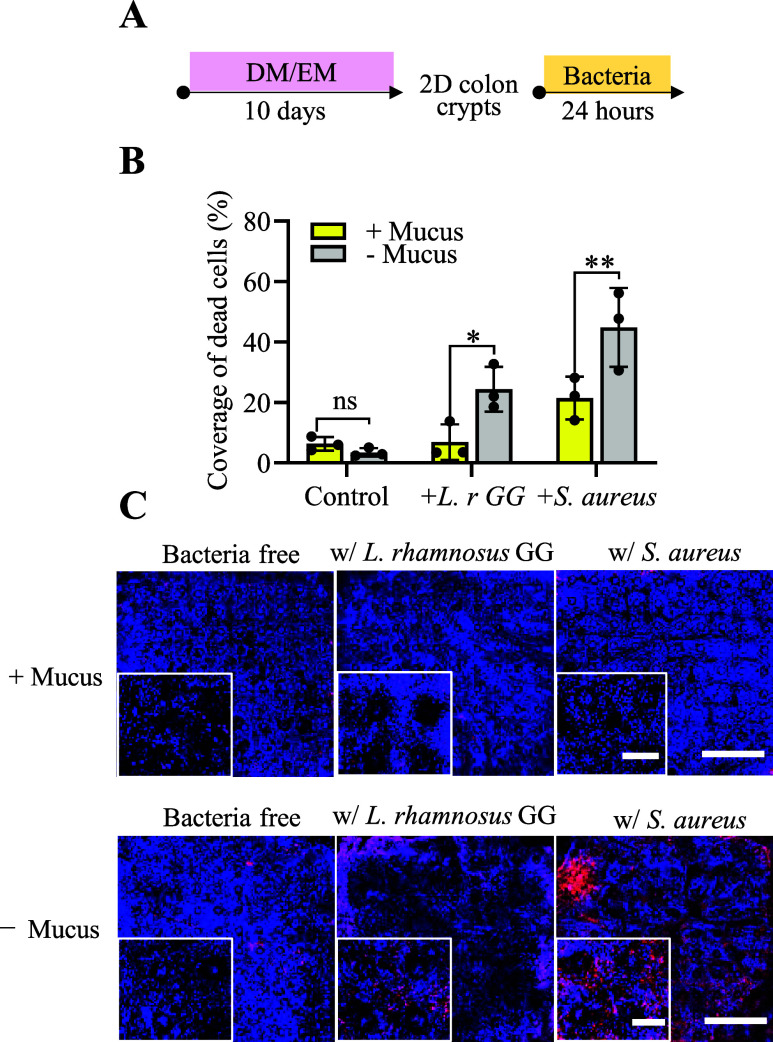
Impact of commensal and pathogenic bacteria on planar crypts with and without a mucus layer. (A) Schematic of the cell culture timeline to form planar crypts for these assays. (B) Percentage propidium iodide positive area relative to that of Hoechst 33342 for the 2 bacteria cocultured with the epithelium with and without a mucus layer. (C) Fluorescence images of planar crypt arrays (*XY*) cocultured with and without *L. rhamnosus* GG or *S. aureus*. Propidium iodide (red) and Hoechst 33342 (blue). Scale bar = 500 μm; inset scale bar = 100 μm. *N* = 3 biological replicates were imaged, i.e., three arrays. Two-way ANOVA multiple comparisons by Tukey. **p < 0.001; *p < 0.05, ns for *P* value >0.05.

*S. aureus* is a common cause of gastrointestinal illness following the consumption of contaminated food.^57^ The pathogenicity of *S. aureus* stems from its multiple toxins, secreted enzymes, and virulence factors, which lead to epithelial cell damage and death by multiple mechanisms.^58–60^ *S. aureus* was added to the luminal reservoir of planar crypts with and without a mucus layer. The planar crypts were incubated for 1 day, and epithelial cell viability was measured (Figure 7A). Controls with and without mucus are expected to have dead cells reflecting the limited lifespan of the terminally differentiated epithelial cells (∼7 days). However, significantly more cells were dead in the presence of *S. aureus* compared to samples without *S. aureus* (*p* = 0.02, Figure 7B). Without a mucus layer and in the presence of *S. aureus*, nearly half of the array was covered by dead cells, significantly more than that for planar crypt arrays with no bacteria or with *L. rhamnosus* (Figure 7C). In all samples, all zones of the planar crypts appeared equally impacted by *S. aureus*. The difference in epithelial cell mortality with and without mucus highlights the functional importance of mucus as a protective barrier. Notably, in the presence of a pathogen under these conditions, the mucus layer mitigated but did not eliminate epithelial cell damage.

## 4 Discussion

In this study, a crypt array with compartmentalized cell types which supported long-term culture of goblet cells and formation of a thick luminal mucus layer was created. *In vivo*, goblet cells are found in all regions of the crypt, i.e., within the crypt as well as along the luminal intestinal surface, with their differentiation being governed by the concentration of growth factors along the crypt long axis.^31^ Complete loss of WNT disrupts epithelial cell renewal.^61^ High WNT activity at the crypt base maintains stemness, while reduced WNT promotes differentiation into secretory lineages, such as goblet cells.^48^ Goblet cell differentiation occurs under reduced rather than absent WNT signaling. The WNT gradient *in vivo* provides spatial signaling for epithelial lineage commitment from stem cells to fully differentiated cells.^62^ A computational simulation of the [WNT] gradient predicted that the WNT concentration did not decrease to zero between the through holes but instead was ≥40% of that at the through hole. Thus, in our system, the radial WNT gradient likely functions as a positional cue that regulates cell differentiation, and the mucus formation is an indirect outcome of goblet cell replenishment from cell renewal. Thus, the optimized array geometry, scaffolding properties, and WRN gradient shape supported goblet cell differentiation, as the cells migrated out to the differentiated cell zone. As a result, the cell ratio formed was 3 ± 1 goblet cells for every 5 ± 2.5 colonocytes for a final proportion of 25% goblet cells and 70–75% colonocytes similar to that *in vivo*.^10^

In this system, goblet cells formed adjacent to the stem/proliferative cell niche consistent with the *in vivo* situation where goblet cell specification is only one division away from a stem cell, i.e., at the crypt base.^63^ As epithelial cells migrate out of the crypt base, they are exposed to a progressive reduction of growth factor concentrations (but not a zero concentration), leading to goblet cell specification. Goblet cell heterogeneity, number and location, mucus layer thickness over time, and mucus barrier function were characterized for the planar crypt array. The biochemical makeup of the mucus changed as the goblet cells migrated from the crypt niche to the luminal surface, as demonstrated by changes in lectin staining at different locations along the crypt long axis. Goblet cells were present throughout the culture period (20 days) most likely due to their continuous renewal from the proliferating progenitor cells in and near the stem cell zone.

Compared to current *in vitro* epithelial models, this newly designed array with flat crypts is a physiological and relevant platform with multiple advantages such as self-renewal, long-term culture, goblet cell replenishment, sustained mucus production, luminal and basal tissue access, primary cell usage, facile imaging, and a diversity of cell types. Prior *in vitro* systems have employed tissue-cultured tumor cells such as Caco-2 or HT-29-MTX cells but these cells with their plethora of mutations do not mimic normal tissue physiology.^64^ In addition, these systems lack a cryptlike organization, goblet cell replenishment, and sustained mucus production. Organoid models have yielded significant insights into intestinal physiology yet do not offer easy luminal access for assay of mucus properties and production.^65,66^ Cells cultured on microphysiological systems have shown enhanced goblet cell differentiation with mechanical deformation or chemical stimulation but often a loose, transient, and filmy layer.^67–69^ In this intestine-mucus MPS, a dense layer of mucus that firmly adhered to the epithelial cells was formed and acted as a barrier to bacterial toxins and bacteria. While the system described in this report is an excellent mimic of the key features of the large intestine, it is noted that mechanical stimulation can enhance replenishment of goblet cells and increase mucus production. Therefore, the addition of shear forces reflecting bowel evacuation using a fecal surrogate^11,44^ would further enhance the model.

## 5 Conclusion

The strengths of this *in vitro* MPS include its use of primary human cells, a physiologic mucus layer produced by human cells, the continual replenishment of goblet cells, and other differentiated cells from primary stem cells allowing long-term culture under a gradient of growth factor concentrations. Additionally, this planar crypt model with a mucus layer offers a clear advantage for its accessibility to both luminal and basal tissue faces, ease of use, and simplicity in imaging relative to more complex 3D systems. This intestine-mucus MPS represents a significant innovation in the development of an *in vitro* model enabling exquisite control of experimental variables for the study of the interplay of the intestinal epithelium, mucus, and microbiota. Future efforts will extensively explore additional mucus attributes, such as biochemical and rheological properties as well as the impact of shear forces upon the epithelium, for example, by using a fecal surrogate.

## Supplementary Material



## Notes

Additional experimental details, materials, and methods, including figures with experimental results (PDF)
