# Supplementary material for: Geometrically Constrained Growth Factor Concentration Favors Enrichment of Goblet Cells and Mucus Formation
Source: ACS Biomater Sci Eng. 2026 Apr 9;12(5):2700–13. doi: 10.1021/acsbiomaterials.5c02107 (PMC13169368; doi:10.1021/acsbiomaterials.5c02107)
Supplement: Supplementary file 1 [file ab5c02107_si_001.pdf]

SUPPORTING INFORMATION

Geometrically constrained growth factor concentration favors enrichment of goblet cells and mucus formation

Cecilia Villegas-Novoa[1], Yuli Wang[1], Hao Wang[1], Ian Jan[1], Christopher E. Sims[1,2], Nancy L. Allbritton[1]*

[1]Department of Bioengineering, University of Washington, Seattle, WA 98195, USA

[2]Department of Medicine, University of Washington, Seattle, WA 98195, USA

∗ Author to whom any correspondence should be addressed. E-mail: nlallbr@uw.edu

Table of Contents

A. Formulation of culture media 2
B. Table S-1. Culture media 2
C. Components: Supplier, catalog number (cat. no.) 3
D. Microfabrication Reagents 3
E. Immunofluorescence (IF) buffer 3
F. Expansion, maintenance, and passage of stem cells on a collagen hydrogel 4
G. Optimization of collagen thickness above the epoxy film 5
H. Crypt array with 350 µm-gap geometry 7
I. Computational modeling of WNT concentration 8
J. Geometry of stem/proliferative zones in 175 µm gap center-to-center array 10
K. SEM images of +/- mucus in 175 µm gap center-to-center microniches array 11

A. Formulation of culture media

In this study, three types of culture media were used. Stem medium (SM) was used to maintain proliferative stem/progenitor monolayers on a collagen hydrogel (1 mg/mL) scaffold in a 6-well plate (Corning, 07-200-83). The expansion medium (EM) was used when the cells were plated on the epoxy film with microholes (luminal and basal side). The differentiation medium (DM) was used only on the luminal side once the cells reached confluency on the film (3 days after seeding). The WRN-conditioned medium was collected from the L-WRN cell line (ATCC, CRL-3276) culture following a published protocol [1].

B. Table S-1. Culture media

Compound	SM	EM	DM
A83-01	500 nM		500 nM
Advanced DMEM/F12	45 vol%	45 vol%	90 vol%
B27	1×	1×	
Fetal bovine serum	10 vol%	10 vol%	10 vol%
Gastrin	10 nM	10 nM	
GlutaMax	1×	1×	1×
HEPES	10 mM	10 mM	10 mM
EGF	50 ng/mL	50 ng/mL	50 ng/mL
NAC	1.25 mM	1.25 mM	
Nicotinamide		10 mM	
PGE2		10 nM	
Primocin	50 μg/mL	50 μg/mL	50 μg/mL
SB202190	3 μM	3 μM	
WRN-conditioned medium	45 vol%	45 vol%	
Y-27632	10 μM*	10 μM	
VIP			330 ng/mL

- EGF: epidermal growth factor
- HEPES: 4-(2-hydroxyethyl)-1-piperazineethanesulfonic acid
- VIP: vasoactive intestinal peptide
- WRN: Wnt-3A, R-spondin 2, Noggin
- NAC: N-acetyl cysteine
- PGE2: Prostaglandin E2

C. Components: Supplier, catalog number (cat. no.)

- (Leu15)-Gastrin-1, human (Anaspec, cat. no. AS-64149)
- A83–01 (Millipore-Sigma, cat. no. SML0788)
- Advanced DMEM/F-12 (ThermoFisher Scientific, cat. no. 12634028)
- B-27 Supplement (50×), serum free (ThermoFisher Scientific, cat. no. 12587001)
- Fetal bovine serum (FBS, R&D Systems, cat. no. S11150)
- GlutaMAX Supplement (ThermoFisher Scientific, cat. no. 35050061)
- HEPES (ThermoFisher Scientific, cat. no. 15630080)
- N-Acetyl-L-cysteine, cell culture reagent, ≥96% (MP Biomedicals, cat. no. 194603)
- Nicotinamide (Millipore-Sigma, cat. no. N0636)
- Primocin (InvivoGen, cat. no. ant-pm-1)
- Prostaglandin E2 (PGE2, Cayman Chemical, cat. no. 14010)
- Recombinant Murine EGF (PeproTech, cat. no. 315–09)
- SB 202190, Free Base, >99% (Selleckchem, cat. no. S-1700)
- Vasoactive intestinal peptide (Anaspec, AS-22872)
- Y-27632 dihydrochloride (ApexBio, cat. no. A3008-200)

D. Microfabrication Reagents

- 1002F epoxy resin (Miller-Stephenson, cat. no. EPON 1002F)
- Acetone (Millipore-Sigma, cat. no. 650501)
- Gamma-butyrolactone (Millipore-Sigma, cat. no. B103608)
- Isopropyl alcohol (Millipore-Sigma, cat. no. 109827)
- Propylene glycol methyl ether acetate (Millipore-Sigma, cat. no. 484431)
- Triarylsulfonium hexafluoroantimonate salts, mixed 50 % in propylene carbonate (Millipore-Sigma, cat. no. 654027)
- Trichloro(octyl)silane (Millipore-Sigma, cat. no. 235725)

E. Immunofluorescence (IF) buffer

- 10% (v/v) 10× PBS
- 0.2% (v/v) Triton X-100
- 0.05% (v/v) Tween-20
- 1 mg/mL bovine serum albumin
- 0.5 mg mL/mL sodium azide

F. Expansion, maintenance, and passage of stem cells on a collagen hydrogel

The human colonic epithelial stem cells were cultured in a monolayer on collagen hydrogel scaffolds (1 mm-thick) using previously published protocols [2, 3]. Isolated crypts from transverse colon tissue of cadaveric donors (RRID: 121 CVCL_ZR41. https://web.expasy.org/cellosaurus/CVCL_ZR41) were plated directly on the collagen hydrogel at a density of 1,000 crypts/well on a 6-well culture plate (9.6 cm^2 of surface area) and cultured with 4 mL stem medium (SM, Table S-1). The medium was changed every 48 hours. The stem cells were cryopreserved at passage 4 (P4) in liquid nitrogen. The cells were karyotyped at P11, and 10 out of 10 spreads displayed normal karyotypes. For this study, cells were expanded from P4 to P8. Expansion of primary colon cells was performed up to P8 for all the experiments. P8-cells from 1 vial were thawed and incubated on a collagen hydrogel surface with 4 mL of SM for 3 days and transferred onto new collagen scaffolds at a 1:2 ratio. After 3 days of incubation, the hydrogels with cells were detached from the 6-well plate and transferred to a 15 mL tube to hydrolyze the collagen with 300μL of collagenase (5000 U/mL) for 10 min at 37°C. The cell pellet was resuspended in EM medium and used directly for each experiment.

G. Optimization of collagen thickness above the epoxy film

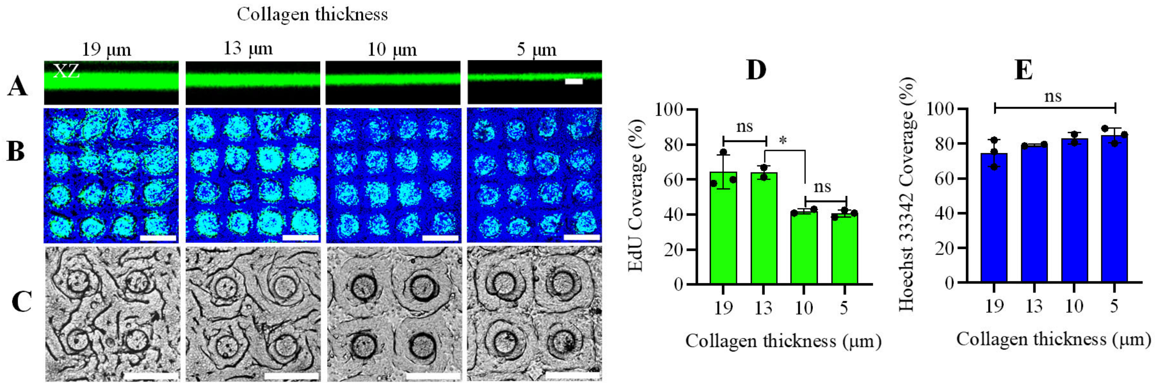

Fig. S-1. Optimization of collagen thickness above the epoxy film. **A.** XZ confocal slices through the collagen layer above the epoxy film. The collagen was labelled with fluorescein. **B.** On day 8 of culture (5 days after planar crypt formation), EdU (24 h pulse) was added to the monolayer of epithelial cells growing above the collagen. Incorporated EdU was then stained (green) and Hoechst 33342 (blue). **C.** Brightfield images of cells growing on the surfaces with different thicknesses collagen layers above the through holes in the epoxy film. **D.** Shown is the EdU coverage for each of the different collagen layer thicknesses. The percentage coverage of EdU was calculated as the array area positive for the fluorescent stain (above an empirically set threshold) divided by the total area of the region examined. Percentage area of the arrays covered by EdU+ cells for the different collagen layers. **E.** Shown is the Hoechst 33342 coverage for each of the different collagen layer thicknesses. The percentage coverage of Hoechst 33342 was calculated as the array area positive for the fluorescent stain (above an empirically set threshold) divided by the

total area of the region examined. For panels D and E, N= 3 biological samples were imaged i.e. 3 arrays and 16 crypts were analyzed for each of the three biological samples. One way ANOVA multiple comparisons by Tukey. **$p<0.001$; *$p<0.05$, not significant (ns) for P value >0.05. A, B, and C: scale bar = 10 µm, 100 µm, and 150 µm, respectively.

H. Crypt array with 350 μm-gap geometry

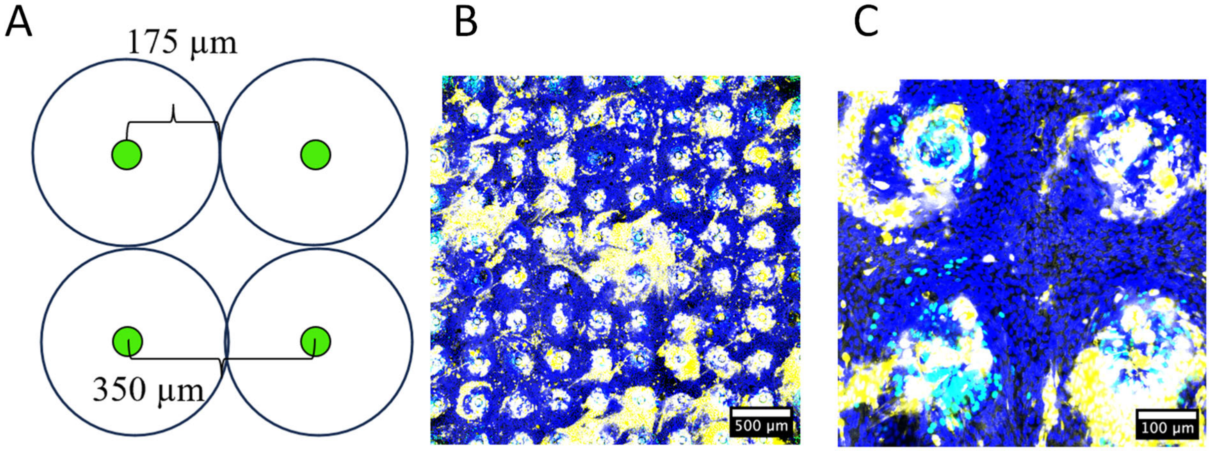

Fig. S-2. Mucus formation on an array with a 350 μm center-to-center through hole gap. A. Geometry of holes. B and C. Confocal microscopy images of sections of an array at different magnifications (Edu=green; Muc2=yellow; Hoechst 33342=blue).

I. Computational modeling of WNT concentration

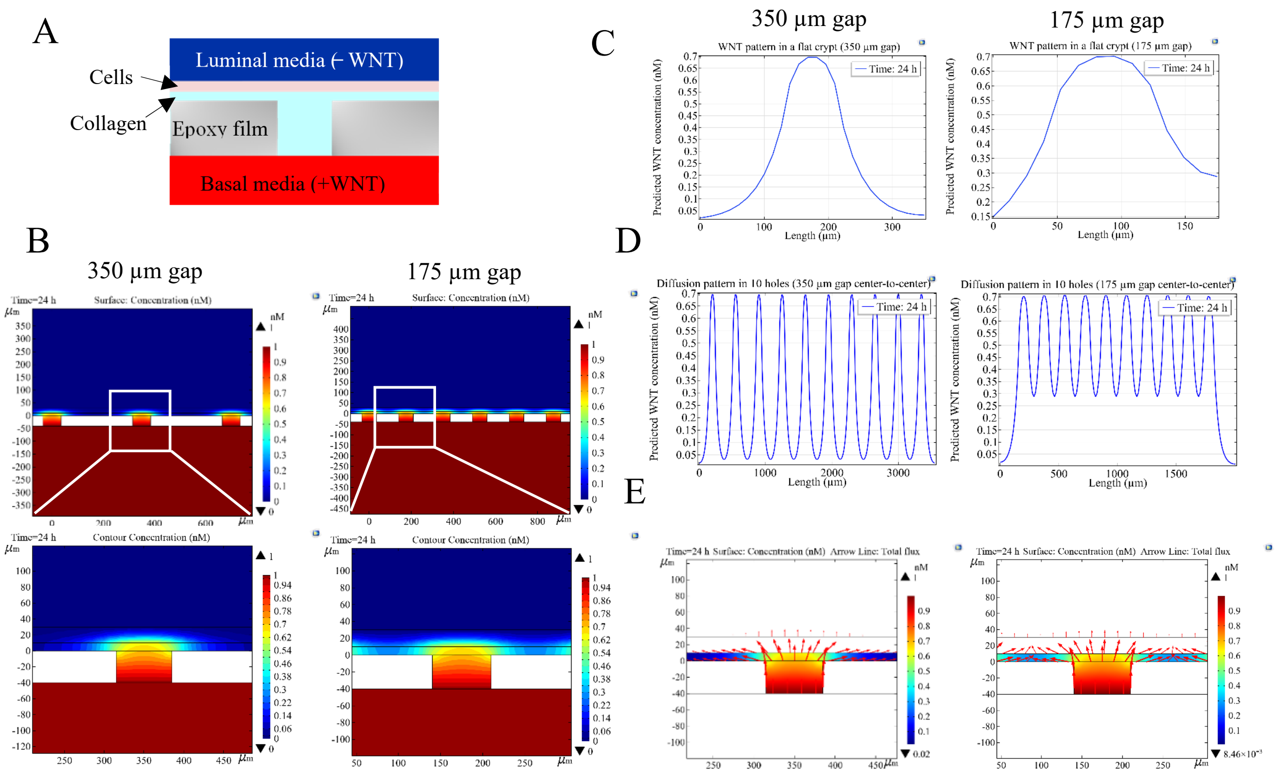

Fig. S-3. Computational modeling of the WNT concentration simulated in COMSOL. A. Schematic of the diffusion model with vertically stacked rectangles (domains) that represent the luminal medium (without WNT at time zero), epithelial cell layer, collagen matrix, patterned epoxy film, and basal medium (with 1 nM WNT at time zero). B. Top panels: Image in the XZ plane of the WNT concentration after 24 h (after equilibrium was achieved) for the 350 and 175 µm gap arrays. Bottom panels: Close-up (XZ plane) of a single through hole showing the diffusion pattern with false coloring to indicate to the WNT concentration [red (1 nM) to blue (0 nM)]. C. Graphic representation of the WNT concentration (near the epithelial cell domain along the Z axis) for a single flat crypt with a center-to-center gap of 350 µm, and a center-to-center gap of 175 µm. D. WNT concentration profiles for 350 and 175 µm center-to-center hole arrays

(10 holes or crypts, near the epithelial cell domain)) E. Concentration of WNT for the two Sdifferent sized arrays with the total flux depicted by arrow size and direction. Directional WNT transport from the basal source through the luminal collagen layer occurred consistent with the formation of a concentration gradient. The diffusion heterogeneity direction was reflected in the flux plot moving from high concentration (bigger arrows) near the basal WNT source, to lower concentrations (smaller arrows) out of the periphery of the crypt niche.

J. Geometry of stem/proliferative zones in 175 µm gap center-to-center array

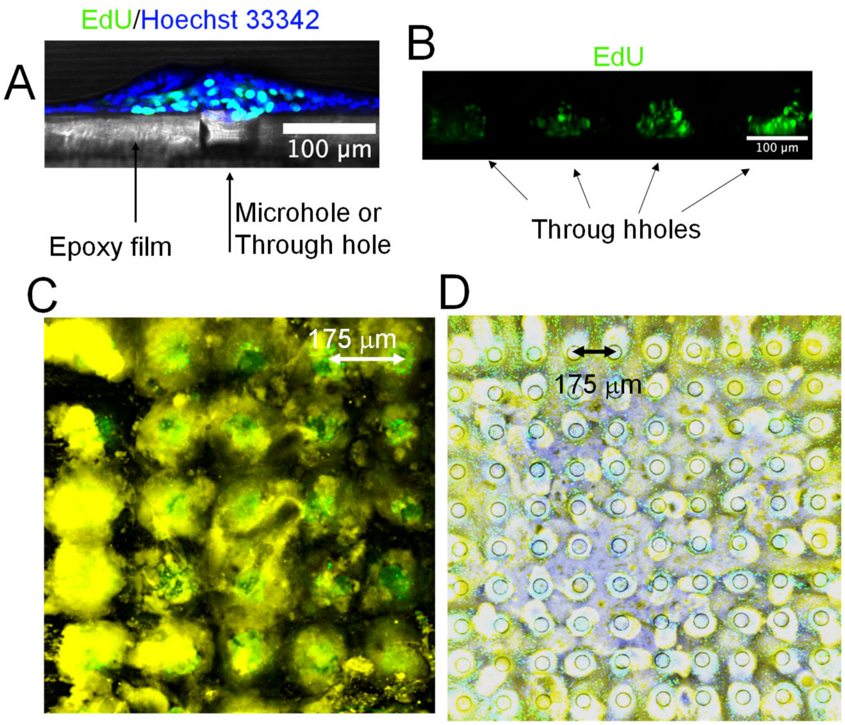

Fig. S-4. Fluorescence images of arrays with a 175 µm center-to-center gap. A. Superimposed brightfield and fluorescence images of a side view through a single through hole. B. Fluorescence image of a side view through 4 through holes. C,D. Fluorescence images of top views of arrays at different magnifications. Yellow=muc2; green=EdU incorporation; blue=Hoechst 33342.

K. SEM images of +/- mucus in 175 µm gap center-to-center microniches array

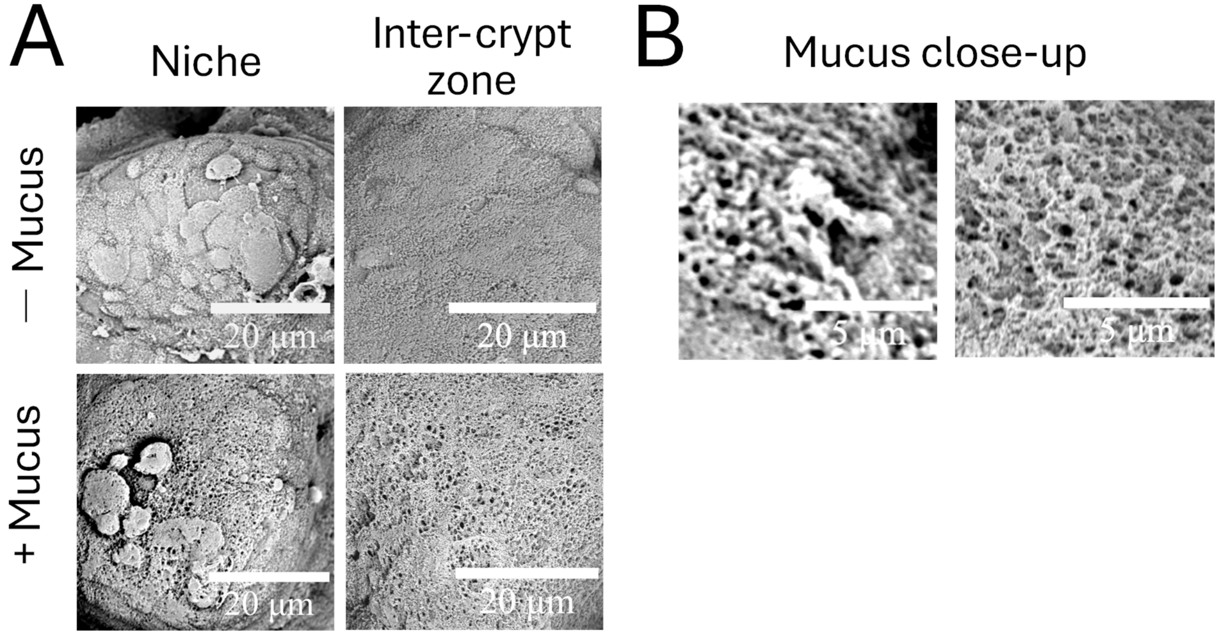

Fig. S-5. Scanning Electron Microscope images of arrays with a 175 µm center-to-center gap. A. Images of the niche (proliferative zone) and inter-crypt zone of the flat crypt with and without mucus. The cobblestone patterning of epithelial cells is visible in the absence of mucus. In the presence of a mucus layer, epithelial cells are not visible due to a porous mucus covering. In the absence of mucus, at the inter-crypt zone, the microvilli on the upper surface of colonocytes are visible. Cells covered with mucus only display the pores within the mucus layer. B. Close-up of the mucus layer above cells. Colonic mucus is shown with a dense homogeneous appearance and tiny pores.

References

[1] H. Miyoshi, T.S. Stappenbeck, In vitro expansion and genetic modification of gastrointestinal stem cells in spheroid culture, Nat Protoc 8(12) (2013) 2471-82.

[2] Y. Wang, M. DiSalvo, D.B. Gunasekara, J. Dutton, A. Proctor, M.S. Lebhar, I.A. Williamson, J. Speer, R.L. Howard, N.M. Smiddy, S.J. Bultman, C.E. Sims, S.T. Magness, N.L. Allbritton, Self-renewing Monolayer of Primary Colonic or Rectal Epithelial Cells, Cell Mol Gastroenterol Hepatol 4(1) (2017) 165-182.e7.

[3] Y. Wang, R. Kim, D.B. Gunasekara, M.I. Reed, M. DiSalvo, D.L. Nguyen, S.J. Bultman, C.E. Sims, S.T. Magness, N.L. Allbritton, Formation of Human Colonic Crypt Array by Application of Chemical Gradients Across a Shaped Epithelial Monolayer, Cell Mol Gastroenterol Hepatol 5(2) (2018) 113-130.

[4] S. Takashima, D. Gold, V. Hartenstein, Stem cells and lineages of the intestine: a developmental and evolutionary perspective, Dev Genes Evol 223(1-2) (2013) 85-102.
